# Human C-peptide Quantitation by LC-MS Isotope-Dilution Assay in Serum or Urine Samples

**DOI:** 10.4172/2157-7064.1000172

**Published:** 2013-03-27

**Authors:** Alexander V Stoyanov, Shawn Connolly, Curt L Rohlfing, Eduard Rogatsky, Daniel Stein, Randie R Little

**Affiliations:** 1 University of Missouri, School of Medicine, Columbia MO, USA; 2 Albert Einstein College of Medicine, Yeshiva University, Bronx NY, USA

**Keywords:** C-peptide/Mass spectrometry, Isotope dilution assay, Ion Exchange chromatography, Sample preparation

## Abstract

In this communication we report a simple and efficient approach to C-peptide quantitation using isotope dilution mass-spectrometry analysis. The method facilitates quantitation of C-peptide levels at least one order of magnitude lower compared to concentration levels achieved with an IDA method reported previously. The improvement was due to more intensive sample preparation procedure that, in turn, makes it possible to increase the sample load without a corresponding increase in matrix effects. We also show the results of a comparison study with a second laboratory using a similar previously reported method for C-peptide quantitation.

## Introduction

C-peptide is a 31-amino acid central part of the pro-insulin molecule. Equal amounts of insulin and C-peptide are released from cleavage of proinsulin by specific beta cell endopeptidases within the pancreatic islets of Langerhans. The exact biological role of C-peptide is not completely understood. C-peptide has generally been considered a byproduct of insulin biosynthesis, but recent data suggest that it may have biological significance [1–3]. In contrast to serum insulin, which is cleared by the liver, C-peptide is cleared at a much slower rate by the kidney and thus represents a more useful indicator of intrinsic insulin secretion (due to 1:1 stoichiometric ratio with insulin) [4–6], Low C-peptide concentrations in plasma can indicate early insulin secretory failure in the preclinical stages of diabetes. Additionally, for diabetes patients who take insulin, C-peptide measurement allows indirect assessment of endogenous insulin production. The reference interval for human C-peptide concentration in plasma is 0.5–10 ng/mL (0.15–3 nmol/L) [7,8]; its concentration in urine is approximately one order of magnitude higher [9,10].

Mass-spectrometric (MS) analysis of C-peptide in processed blood (plasma samples) was first performed in 1996 [7] and was one of the first successful applications of the MS isotope dilution assay (IDA) for quantitative analysis of endogenous peptides. The authors used reversed phase chromatography in LC-MS quantitation, and employed solid phase extraction for sample preparation. The same general principle was utilized by other investigators who contributed to further method development [10–16], mainly aimed at increasing analysis throughput.

To overcome the negative effects of poor ionization efficiency for C-peptide in MS analysis, a method that utilized two-dimensional reversed phase-reversed phase chromatography was introduced in 2006 [14,15]. A relatively small peak heart-cut fraction, less than 0.3mL containing the C-peptide peak was eluted using a shallow linear gradient during the first dimension separation; this fraction was then transferred to a second dimension reversed phase column prior to MS analysis. Two-dimensional chromatography was thus realized without complete sample mapping [17]. Although, in each dimension the same separation mechanism based on hydrophobic interaction was used, the signal to noise ratio was greatly improved, presumably due to the fact that different hydrophobic interaction stationary phases were employed sequentially using different ion-pairing agents [15], which is resulted in different column selectivities. Importantly, the most abundant fragment ion of C-peptide is Y1 *[m/z* 147.1], which represents a yield of only 1.5% using collision induced fragmentation (the standard fragmentation fragmentation technique implemented in multiple-reaction monitoring MRM)). Therefore, to achieve optimal sensitivity, MRM mode was avoided and selective-ion monitoring mode was used to quantitate the precursor ions of C-peptide and its stable labeled internal standard. The resulting decreased selectivity of the mass spectrometry analysis was compensated for by the improved 2D LC separation.

An alternative approach to sample preparation has been reported recently where the authors proposed the use of ion exchange chromatography [11], The main idea of this approach was to take advantage of the high acidity of the peptide of interest to perform the appropriate purification step. High C-peptide acidity results from the fact that there are no positive charged amino acid residues in the peptide sequence except for the N-terminal amino group. As result, C-peptide is not retained on cation exchanger stationary phases even under strong acidic conditions. This property facilitated the use of an isolation scheme that utilized the negative absorption of C-peptide on cation exchanger resin; the detailed conditions were selected based on the theoretical analysis of C-peptide electric charge vs. pH curve [18–21].

In the present paper we report an improved version of this previously developed method [11] which allows for measurements of lower C-peptide concentration in biological samples. The sample preparation procedure is summarized in the table 1.

The general purification scheme consisted of three steps and started with methanol precipitation, where methanol was added to serum samples (mixed with a standard) in ratios up to 4:1 (typically, 40μL of IS, 100ng/mL, were mixed with 160μL of serum sample then 0.8 mL of methanol was added). After centrifugation the supernatant was immediately applied to SepPak C18 disposable cartridges (Millipore, Billerica, MA). The methanol concentration of the effluent was then reduced to 20% and the pH brought to 3.95 by ammonium formate buffer prior to cation-exchange purification using HiTrap HP SP cartridges (GE Healthcare); for this step up to 3ml of effluent was applied to the SP column. The fourth step, anion-exchange purification with HiTrap HP QP, was not used for routine analysis but was successfully employed for large sample pre-concentration in cases where samples had very low C-peptide concentrations. A concentration factor of 10–20 was achieved with recoveries ≥ 75%. After purification, the samples were subjected to LC-MS analysis as previously described [11]. The HPLC procedure included 15 min separation in an acetonitrile gradient; its total duration lasted 35 minutes including two wash cycles.

**Figure 1:**
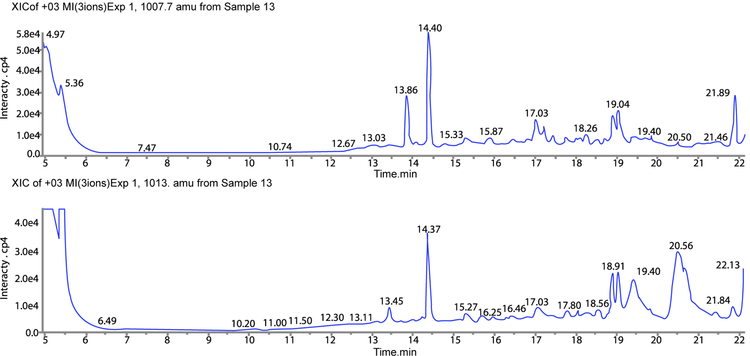
Purified C-peptide MS characterization. A: SIM for 1007.7 (native) and B: 1013.0 standard are shown. Retention time for C-peptide is 14.7min.

**Figure 2:**
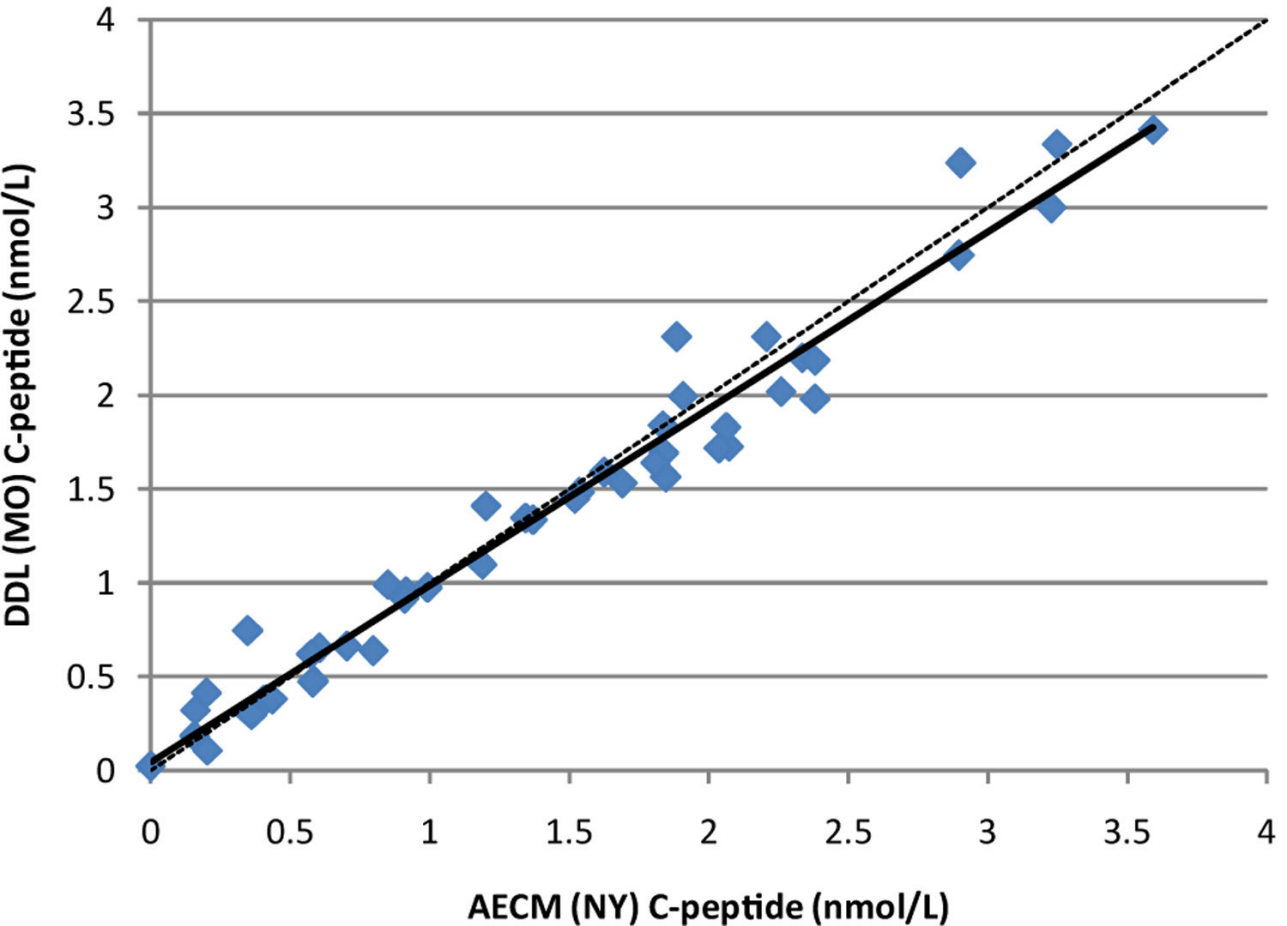
Method comparison between the MO and NY laboratories; n=47, y=0.941x+0.047, R^2^=0.9647. Solid line is the regression line; dashed line is y=x.

The three-stage procedure described was very effective in achieving C-peptide concentrations suitable for quantitation using LC-MS (Figures 1–2). C-peptide tolerates very high concentrations of alcohol which facilitated precipitation of most impurities in the first stage. However, the high alcohol concentration resulting from the first stage requires further sample dilution to be performed prior to the next ion-exchange purification stage where methanol concentration should not exceed 20 % in order to avoid significant change in the acid-base chemistry/ion-exchange interaction. Since the total volume we could apply to LC-MS is limited (in our case, the sample loop had a volume of 2ml) some compromise was required, (Table 2). We found that mixing four parts methanol to one part serum provided optimal results.

**Figure 3:**
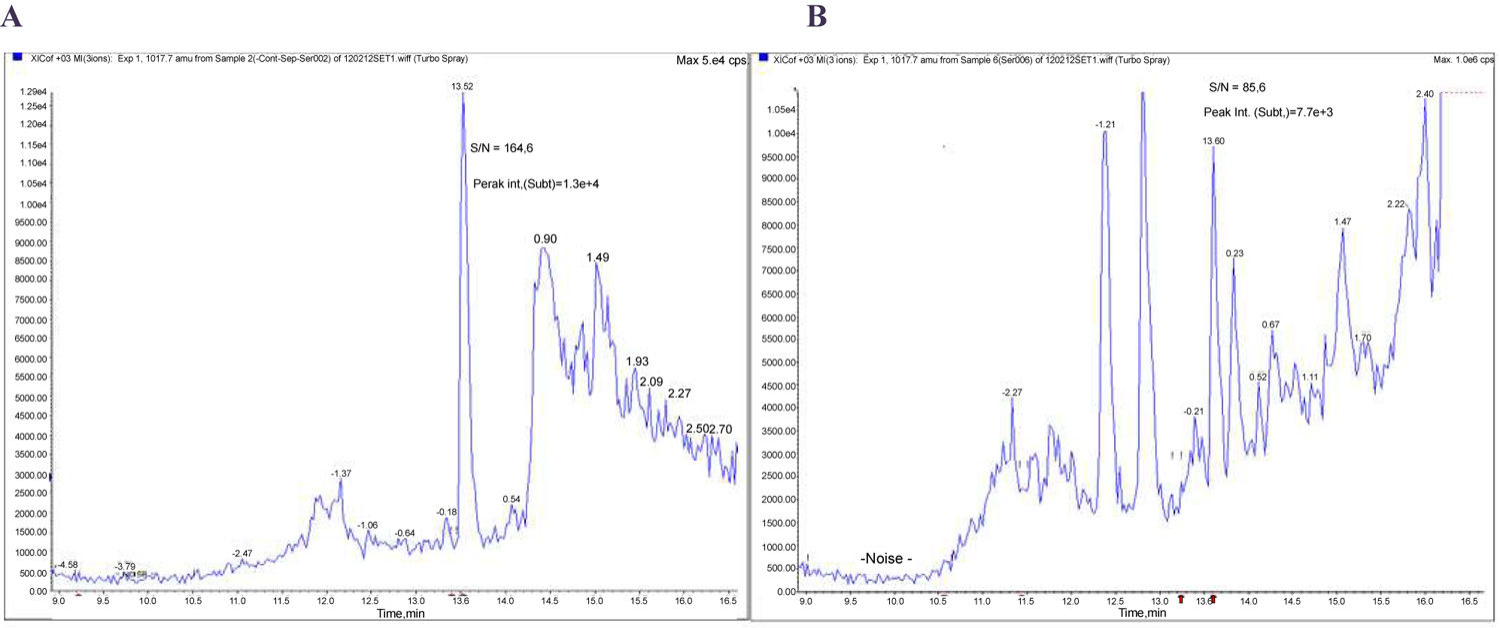
Comparison of C-peptide internal standard (IS) mass spectrograms for pure IS (A) and IS mixed with the serum sample (B). A: 50 pg of pure stable isotope labeled C-peptide (retention time 13.5 min, S/N 165) B: 50 pg on column of the same isotope as in A spiked in serum (retention time 13.6 min, S/N 86). The lower signal intensity observed for IS (Mw/z=1017.7) mixed with serum is due to both matrix effect and analyte losses during purification steps.

The results of C-peptide measurements in human serum by the method described here were compared with those of a two-dimensional (2D) reversed phase chromatography method of Rogatsky et.al. The modified method was performed at the Diabetes Diagnostic Laboratory (DDL), Columbia, MO. The 2D method was performed at the Albert Einstein College of Medicine (AECM), Bronx, NY [15,16]. Forty-seven serum samples were analyzed by both methods; the results are shown in Figure 3. For each sample, X and Y represent the values obtained in the New York and Columbia, MO, laboratories, respectively. The two methods showed excellent correlation (R^2^=0.9647); results from the MO laboratory were slightly lower (~4%) overall compared to results from the NY laboratory.

The improved method for LC-MS quantitative analysis of C-peptide in human plasma as well in urine described here facilitates quantification of C-peptide in patient samples with C-peptide concentrations as low as 20pg/ml, or 7pMol/l. The sample preparation procedure increased analytical sensitivity at least 10x compared to results reported previously. The method is fast and robust and allows for high throughput. The sample preparation procedure does not employ traditional concentration techniques such as ultrafiltration and lyophilization which are very time consuming and normally result in considerable sample loss. Method comparison data demonstrated excellent correlation with another previously described LC-MS method.

## Figures and Tables

**Table 1:** C-peptide purification scheme. Methanol precipitation was followed by centrifugation; the supernatant was then loaded onto C18 SepPak cartridge. The effluent was then applied to the ion exchanger after dilution of the methanol concentration and pH adjustment.

Stage	Procedure	Conditions
1	precipitation with methanol	v/v (4:6–8:2) followed by centrifugation at 12 g, 7 min
2	SepPak C18	in presence of methanol >70%
3	HiTrap SP HP	Methanol<20%, pH 2.95
4	HiTrap Q HP	Methanol<20%,sample application pH 7.0, elution 0.4% of formic acid (pH~2)

**Table 2:** Final sample dilution factor as a result of two step purification. After methanol precipitation, additional sample dilution is caused by the necessity of reducing the content of organic solvent to the desired concentration (20% in this particular case, s=0.2). This “double dilution” results in very fast analyte content decline per sample volume, as given by the relationship: *f=(1−S)(s/S)*. The values are reported without taking into account a correction for volumes non-additivity.

Added organic solvent content, S, v/v	Approximate final dilution factor
0.2 (20%)	1.25 (80%)
0.3 (30%)	2.15 (47%)
0.4 (40%)	3.3 (30%)
0.5 (50%)	5 (20%)
0.6 (60%)	7.5 (13%)
0.7 (70%)	12 (8.6%)
0.8 (80%)	20 (5%)
0.9 (90%)	45 (2.2%)

## References

[1] Haidet J, Cifarelli V, Trucco M, Luppi P (2009) Anti-inflammatory properties of C-Peptide. Rev Diabet Stud 6: 168–179. 20039006 10.1900/RDS.2009.6.168 PMC2827269

[2] Sun W, Gao X, Zhao X, Cui D, Xia Q (2010) Beneficial effects of C-peptide on renal morphology in diabetic rats. Acta Biochim Biophys Sin (Shanghai) 42: 893–899. 21106770 10.1093/abbs/gmq100

[3] Chima RS, LaMontagne T, Piraino G, Hake PW, Denenberg A (2011) C-peptide, a novel inhibitor of lung inflammation following hemorrhagic shock. Am J Physiol Lung Cell Mol Physiol 300: L730–739. 21398498 10.1152/ajplung.00308.2010 PMC3094028

[4] Steiner DF, Cunningham D, Spigelman L, Aten B (1967) Insulin biosynthesis: evidence for a precursor. Science 157: 697–700. 4291105 10.1126/science.157.3789.697

[5] Eaton RP, Allen RC, Schade DS (1983) Hepatic removal of insulin normal man: dose response to endogenous insulin secretion. J Clin Endocrinol Metab 56: 1294–1300. 6341392 10.1210/jcem-56-6-1294

[6] Polonsky KS, Given BD, Hirsch L, Shapiro ET, Tillil H, (1988) Quantitative study of insulin secretion and clearance in normal and obese subjects. J Clin Invest 81: 435–441. 3276729 10.1172/JCI113338 PMC329588

[7] Kippen AD, Cerini F, Vadas L, Stöcklin R, Vu L, (1987) Development of an isotope dilution assay for precise determination of insulin, C-peptide, and proinsulin levels in non-diabetic and type 2 diabetic individuals with comparison to immunoassay. J Biol Chem 272: 12513–12522. 10.1074/jbc.272.19.12513 9139702

[8] Clark PM (1999) Assays for insulin, proinsulin (s) and C-peptide. Ann Clin Biochem 36: 541–564. 10505204 10.1177/000456329903600501

[9] Horwitz DL, Rubenstein AH, Katz Al (1977) Quantitation of human ß-cell function by immunoassay of C-peptide in urine. Diabetes 26: 30–35. 318625 10.2337/diab.26.1.30

[10] Fierens C, Stockl D, Baetens D, Leenheer A, Thienpont L (2000) Quantitative analysis of urinary C-peptide by liquid chromatography-tandem mass spectrometry with a stable isotopically labeled Internal standard. J Chromatogr A 896: 275–278. 11093662 10.1016/s0021-9673(00)00717-2

[11] Stoyanov AV, Rohlfing CL, Connolly S, Roberts ML, Nauser CL, (2011) Use of cation exchange chromatography for human C-peptide isotope dilution-mass spectrometric assay. J Chromatogr A 1218: 9244–9249. 22098929 10.1016/j.chroma.2011.10.080 PMC5089808

[12] Fierens C, Thienpont LM, Stockl D, Willekens E, De Leenheer AP (2000) Quantitative analysis of urinary C-peptide by liquid chromatography-tandem mass spectrometry with a stable isotopically labelled internal standard. J Chromatogr A 896: 275–278. 11093662 10.1016/s0021-9673(00)00717-2

[13] Fierens C, Stockl D, Baetens D, Leenheer AP, Thienpont LM (2003) Standardization of C-Peptide measurements in Urine by Method Comparison with Isotope-Dilution Mass Spectrometry. Clin Chem 49: 992–994. 12766009 10.1373/49.6.992

[14] Rogatsky E, Balent B, Goswami G, Tomuta V, Jayatillake H, (2006) Sensitive quantitative analysis of C-peptide in human plasma by 2-dimensional liquid chromatography-mass spectrometry isotope-dilution assay. Clin Chem 52: 872–879. 16556683 10.1373/clinchem.2005.063081

[15] Rogatsky E, Tomuta V, Cruikshank G, Vele L, Jayatillake H, (2006) Direct sensitive quantitative IC/MS analysis of C-peptide from human urine by two dimensional reverse phase/reverse phase high-performance liquid chromatography J Sep Sci 29: 529–537. 16583691 10.1002/jssc.200500369

[16] Rogatsky E, Tomuta V, Jayatillake H, Cruikshank G, Vele L, (2007) Trace LC/MS quantitative analysis of polypeptide biomarkers: impact of 1-D and 2-D chromatography on matrix effects and sensitivity. J Sep Sci 30: 226–233. 17390616 10.1002/jssc.200600250

[17] Stoyanov A (2012) IEF-based multidimensional applications in proteomics: toward higher resolution. Electrophoresis 33: 3281–3290. 23097021 10.1002/elps.201200221

[18] Castagnola M, Rossetti DV, Corda M, Pellegrini M, Misiti F, (1998) The pH dependence of predictive models relating electrophoretic mobility to peptide chemico-physical properties in capillary zone electrophoresis. Electrophoresis 19: 2273–2277. 9788308 10.1002/elps.1150191304

[19] Stoyanov AV, Righetti PG (1999) Dissociation of polyvalent electrolytes. J Chromatogr A 853: 35–44. 10486710 10.1016/s0021-9673(99)00455-0

[20] Righetti PG, Stoyanov A, Zhukov M (2001) The Proteome Revisited: Theory and Practice of All Relevant Electrophoretic Steps. Elsevier Amsterdam.

[21] Oran PE, Jarvis JW, Borges CR, Nelson RW (2010) C-peptide microheterogeneity in type 2 diabetes populations. Proteomics: Clin Appl 4: 106–111. 21137020 10.1002/prca.200800249 PMC3761384

